# The Marvels of Surgery

**Published:** 1899-10

**Authors:** 


ARTICLE XIV.

### THE MARVELS OF SURGERY.

The feats of modern surgeons are indeed truly marvelous and wonderful. There is nothing they will not attempt, to save life or render it easier for the sufferer. The defects of nature, as weil as the results of accidents, are set right, says the *St. Louis Globe-Democrat.* A girl of eight years, though of normal physique, had no inteligence. Her parents had noticed this from her first year, and, though everything had been done for her in the way of education, she did not know the letters of the alphabet, nor could she talk intelligently. A surgeon, who was consulted, gave as his opinion that the brain had ceased to develop, owing to the premature coalesence of the bones of the skull. He removed several small pieces of bones from the top of the head, and by thus relieving the pressure on the brain gave it room to expand. Within twenty-four hours of the operation its effects were seen. The child cried for its parents, took notice of everything, and within eight days could talk well and amuse herself intelligently. In cases of injury to the skull the pieces of crushed-in bone are removed and the vacant spaces covered by silver plates

The modern surgeon, indeed, can replace any part of the human body which may be injured, and rig up an entirely new set of limbs. A housemaid in a hotel was struck in the face by a descending lift and her nose severely injured. Attempts to patch up the damaged organ failed, and it was determined to make a new nose. A black-bird was killed, and before its body was cold its breastbone was fixed to the woman's face, and what remained of the old skin was drawn over it. The strange substitute knitted itself to the face, the operation being a complete success.

The operation of rhinoplasty is a very common one at Heidelberg, as the students there have an ugly habit of slashing each other's noses in their famous duels. A flap of skin is almost detached from the forehead and brought down over the nose which has been almost destroyed. The skin is then stitched down on either side of the nose, and in time becomes grafted thereto, Skin-grafting, indeed, is quite common in cases of severe burns. Small strips of skin are taken from the untouched parts and cut into small pieces and distributed over the raw surface. In time they take root and grow and spread until they completely cover the place. The skin of frogs, recently killed for the purpose, is frequently used where human cuticle cannot be conveniently obtained.

A month or two ago a doctor was called in to attend a boy whose ear had been completely bitten off by a vicious horse. The surgeon determined to try and replace the ear, as failure to do so could not result in a worse deformity. The missing ear was duly found and handed to the doctor, who was then engaged in bathing the severed part in warm water. He had neither instruments nor dressings with him, and as the half hour's delay to obtain them would have been fatal to success, he stitched the ear in its place again with a common needle and thread. This was followed by antiseptic treatment, and in six weeks the ear had become reunited to the head again and completely healed, leaving no scars. Even had this been a failure, an aural appendage, made of waxy composition and an exact facsmile of the other ear, could have been made and fixed. In some cases it has been necessary to remove the tongue, but by raising the floor of the mouth and thus in some way filling the place of the missing organ the patient has been enabled to speak almost perfectly.

The fitting of glass eyes is well known and the complete destruction of the jawbone has no terrors for the modern surgeon. The crushed bone is removed and a piece of silver or aluminum, the exact shape of the lost jaw, fitted in its place. After this has become firmly fixed, teeth can be fitted to it. If a man's throat is defective the operation of trachetomy—the insertion of a silver tube in wind-pipe with an orifice opening to the throat—provides him with a new breathing apparatus. Artificial legs and arms are now so perfect that with them a man can walk, skate and even cycle. There is a story also of a man who, injuring his spine in a railway accident, was fitted with a steel casing for his backbone, and so enabled to walk and ride.

Bones destroyed by accident or decayed by disease are placed by others taken from animals or amputated limbs. A bone in a man's foot was attacked by caries, which gradually ate the bone away. A lamb's shank was procured, the bone cleaned, boiled in carbolic acid, pounded into a powder and made into a paste. The foot was opened and all the decayed parts of the bone scraped away, simply leaving a hollow shell. This was packed with the paste of the lamb's shank and the wound stiched up. It speedily healed and the free use of the foot was soon recovered.

It was during the attack on Santa Cruz in 1779 that Nelson lost his arm. He was shot through the right elbow, and after binding it up with a handkerchief, he continued to direct the fighting. When the battle was over Nelson was examined by the surgeon, who decided amputation was necessary. The sea was rough, the cockpit badly lighted, and chloroform, of course, unknown. In tying up the separated arteries the surgeon accidentally bound up the median nerve also. This "unlucky circumstance," as Nelson called it, was productive of intense suffering to the gallant admiral, but the foremost surgeons in England could not devise any treatment to relieve him. To a modern surgeon the course would be straightforward and simple. The wound would be opened and the nerve released from the silk ligature.

Where nerves have been accidently cut during an operation or by glass, they have been successfully joined together again. An artisan, falling from a height, severely hurt his right arm.

It was operated upon, and though specifically successful, the arm wasted and was quite useless, preventing the man from following his trade. It was thought that a nerve had been severed either by the surgeon during the operation or torn in the fall. The arm was opened, and this was found to be the case. A rabbit was procured rendered unconscious and the two sciatic nerves extracted and stitched to the ends of the severed nerve in the man's arm. Within a month the man had recovered the full use of his arm, which also regained its normal size and appearance, and he was soon able to follow his trade again.

Even to the heart itself has been applied the ordinary treatment of wounds. A patient was brought to a hospital, stabbed to the heart. The surgeon laid bare the heart, and finding the sight of the wound on the right side, calmly stiched it up. The heart beat most violently during this operation, but afterward quieted down and the patient recovered from what has usually been regarded as a fatal wound.

Though these performances sound marvelous to modern ears, the civilization of prehistoric times furnishes instances quite as remarkable. The operation of trephining —the cutting out of a piece of the skull which has been crushed in and replacing it by a silver or gold plate— which is one of the most delicate known to modern surgery, was performed by the ancient Incas of Mexico and

Peru. In the Smithsonian Institute there are nineteen skulls of prehistoric date which bear traces of this operation having been performed on them.

---

### Monthly Summary.

